# A Rare Case of Bacterial Endocarditis Leading to Aortic Valve Insufficiency and Presenting as Myocardial Infarction

**DOI:** 10.7759/cureus.104247

**Published:** 2026-02-25

**Authors:** Ganesh Jayadevappa, Sandeep Srivastav

**Affiliations:** 1 Anesthesiology, Aster Hospital, Dubai, ARE; 2 Cardiology, Aster Hospital, Dubai, ARE

**Keywords:** aortic valve replacement, bacteremia, endocarditis, methicillin-resistant staphylococcus aureus (mrsa), myocardial infarction

## Abstract

Infective endocarditis (IE) is a life-threatening condition that can lead to valvular destruction, systemic embolization, and multiorgan failure. While IE typically presents with fever and murmur, acute myocardial infarction (MI) is a rare initial manifestation, particularly in postpartum women. We report the case of a 34-year-old postpartum female who presented with chest pain and hypotension. Electrocardiography showed ST-segment changes suggestive of anterolateral MI. Laboratory tests revealed elevated troponin, leukocytosis, and methicillin-resistant *Staphylococcus aureus* bacteremia. Coronary angiography identified thrombotic occlusions, and echocardiography showed a mobile vegetation on the aortic valve with severe regurgitation. She underwent angioplasty, targeted antibiotics, and emergency valve replacement. Intraoperative findings confirmed extensive valve destruction. Postoperative recovery was uneventful. This case underscores the importance of considering IE in young patients with MI-like symptoms and systemic infection. Early echocardiography, blood cultures, and timely surgical intervention are critical for survival in such complex presentations.

## Introduction

Infective endocarditis (IE) is a critical and potentially fatal condition involving an infection of the inner lining of the heart, particularly affecting the heart valves. It is associated with high morbidity and mortality, especially when caused by aggressive pathogens such as *Staphylococcus aureus*, including its methicillin-resistant strains (MRSA) [1]. The classical presentation includes fever, a new or changing murmur, and signs of systemic embolism. However, IE can occasionally present with atypical features such as acute coronary syndrome (ACS) or myocardial infarction (MI), especially when embolic vegetations obstruct coronary arteries [2].

Pregnancy and the postpartum period are states of immune modulation, rendering women more susceptible to infections, including endocarditis, particularly in the presence of risk factors such as recent cesarean section or bacteremia [3]. Although postpartum IE is rare, immune suppression and invasive delivery procedures can disrupt mucosal barriers, allowing opportunistic infections to spread systemically [4].

Early diagnosis relies on microbiological and echocardiographic evidence, guided by the modified Duke criteria. Key indicators include positive blood cultures and echocardiographic signs of endocardial involvement [5]. Our report describes a rare case of MRSA endocarditis in a postpartum woman presenting as MI, emphasizing the diagnostic challenge and the necessity of rapid, multidisciplinary management. This case report follows the CARE (CAse REport) guidelines [6].

## Case presentation

History of presentation and past medical history

A 34-year-old primiparous lactating female, three months postpartum following a cesarean section, presented to the emergency department with complaints of chest pain, dyspnea, easy fatigability, dizziness, intermittent fever, and sweating for the past 10 days. She denied any prior history of cardiovascular or systemic illness. The chest pain was sudden in onset, retrosternal, and radiating to the left arm. No history of rheumatic heart disease, dental procedures, or recent hospitalization was noted apart from delivery.

Examination findings

On admission, the patient was ill-looking, febrile, and hypotensive with a blood pressure of 83/50 mmHg. Heart rate was 109 beats/minute, respiratory rate was 22 breaths/minute, and oxygen saturation was 94% on room air. Her body mass index was 37.6 kg/m². Cardiovascular examination revealed audible S1 and S2 without murmurs. Bilateral basal crepitations were heard on auscultation. The abdomen was soft with normal bowel sounds.

Investigations

Initial laboratory workup revealed elevated inflammatory markers: C-reactive protein at 454 mg/L (normal range: <5 mg/L), procalcitonin at 5 ng/mL, and leukocytosis (white blood cell count: 19.0 × 10⁹/L). Troponin-T was markedly elevated at 1,433 pg/mL, suggestive of myocardial injury. Blood cultures isolated MRSA. Renal parameters were deranged with serum creatinine of 2.08 mg/dL and urea of 80.9 mg/dL. Liver function tests showed transaminitis: serum glutamic oxaloacetic transaminase of 636 U/L, serum glutamic pyruvic transaminase of 600 U/L, and elevated bilirubin levels.

**Figure 1:**
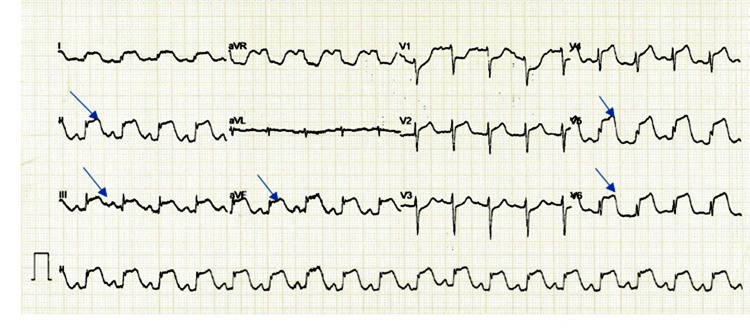
Electrocardiogram showing ST‑segment elevation.

Electrocardiogram (ECG) revealed ST-segment changes in leads II, III, aVF, and V4-V6, suggestive of inferolateral MI (Figure 1).

**Figure 2:**
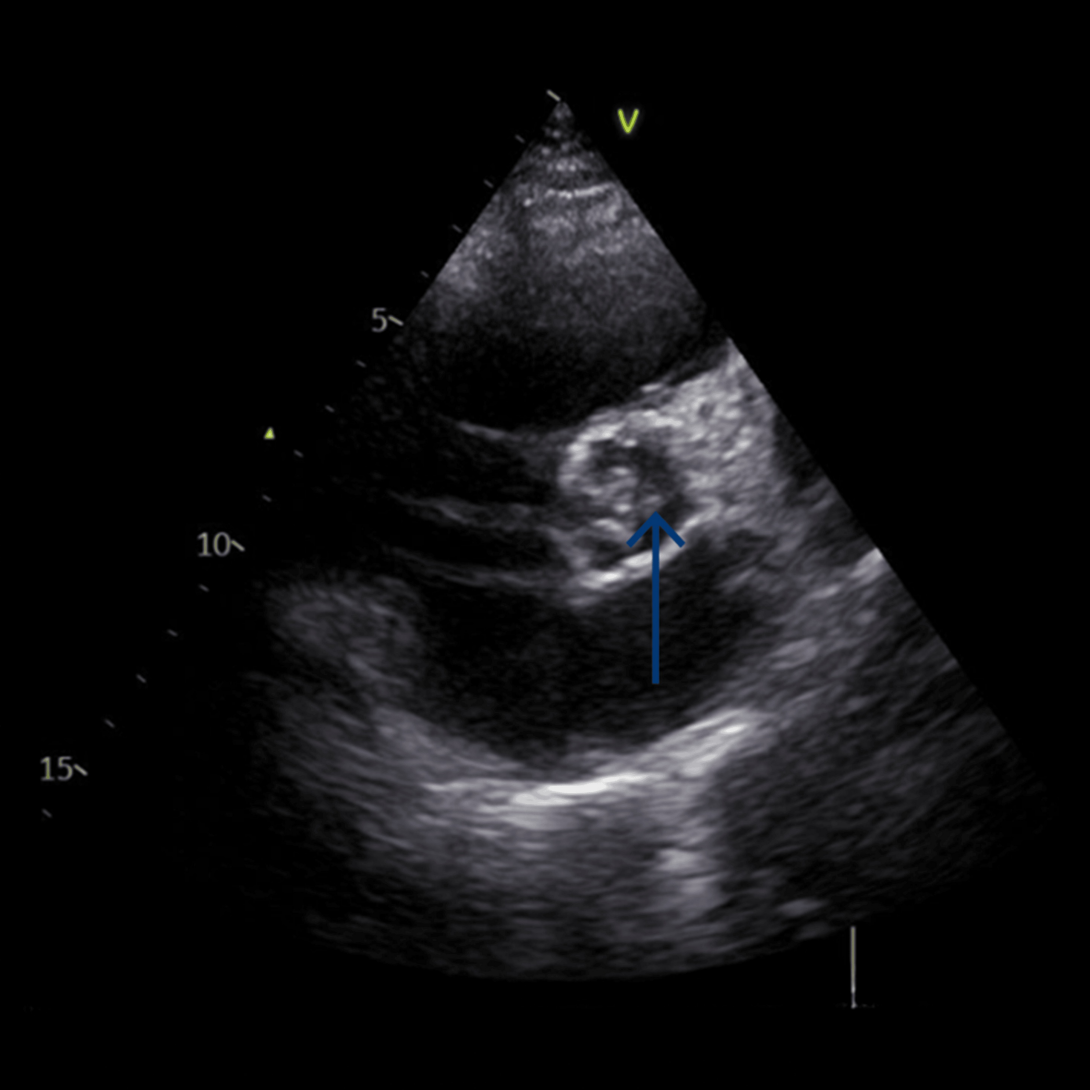
Transoesophageal echocardiography showing aortic valve vegetation.

Transthoracic echocardiography showed a mobile, pedunculated vegetation measuring 1.3 × 0.5 cm attached to the aortic valve with severe eccentric aortic regurgitation, severe tricuspid regurgitation, and pulmonary arterial hypertension (right ventricular systolic pressure: 75 mmHg) (Figure 2).

**Figure 3:**
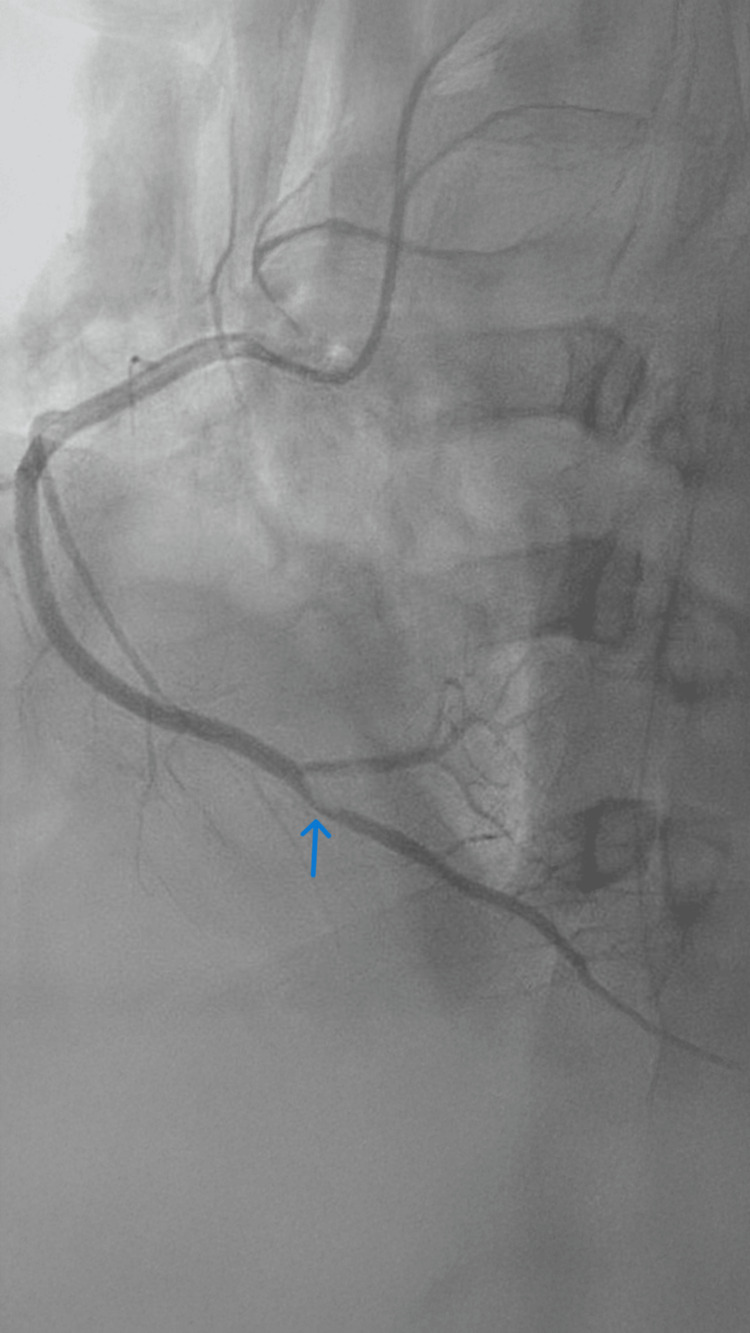
Coronary angiogram showing thrombus.

Coronary angiography demonstrated thrombotic occlusion of the posterior descending artery and posterolateral branch (Figure 3).

**Figure 4:**
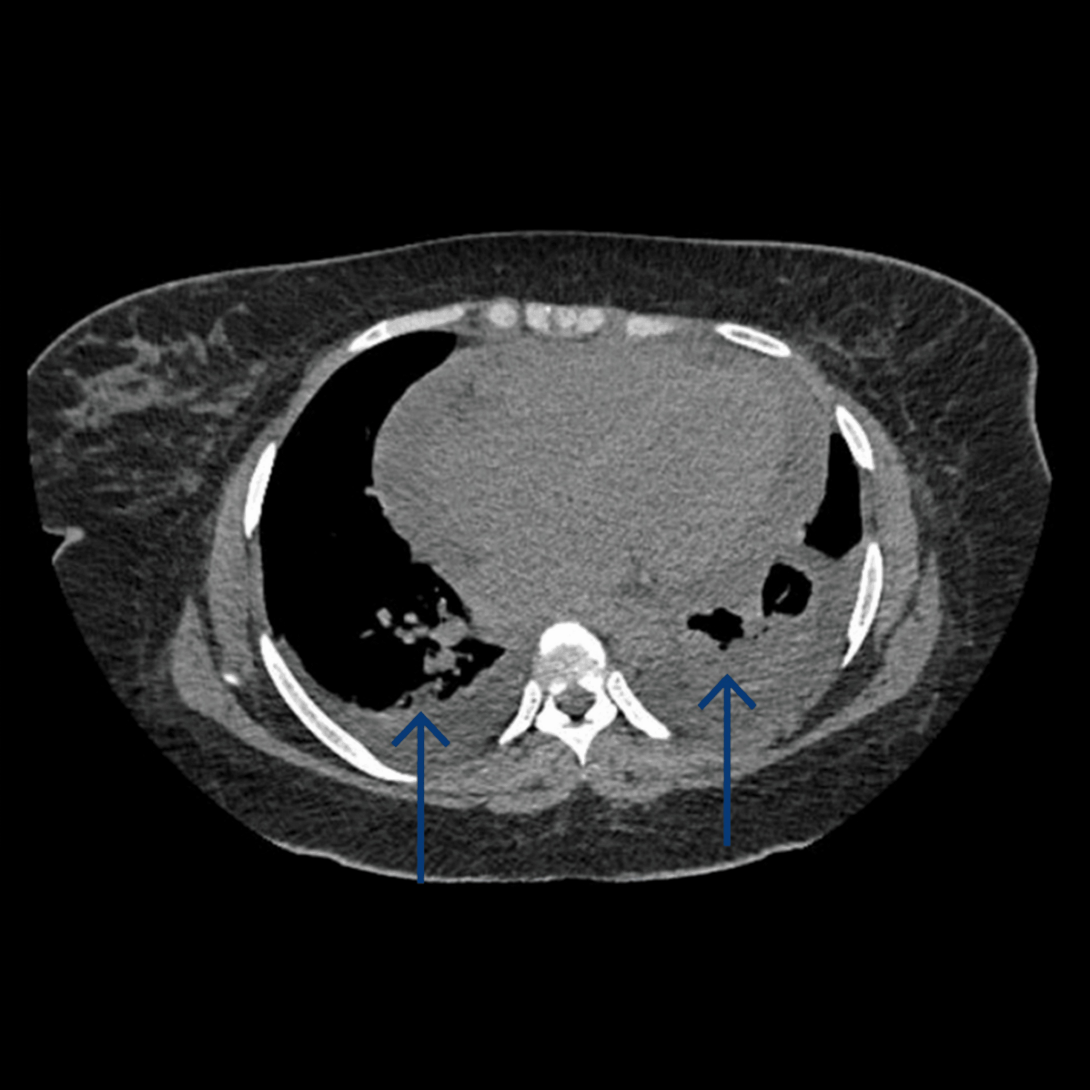
CT of the chest showing cardiomegaly and pulmonary edema.

CT of the chest revealed cardiomegaly, basal consolidations, smooth interlobular septal thickening, and bilateral pleural effusions, consistent with acute pulmonary edema (Figure 4).

Relevant laboratory investigations on admission are summarized in Table 1, highlighting systemic inflammation, myocardial injury, renal and hepatic dysfunction, and microbiological confirmation of MRSA IE.

**Table 1:** Summary of laboratory investigations on admission. WBC = white blood cell; CRP = C-reactive protein; SGOT = serum glutamic oxaloacetic transaminase; AST = aspartate aminotransferase; SGPT = serum glutamic pyruvic transaminase; ALT = alanine aminotransferase; MRSA = methicillin-resistant *Staphylococcus aureus*

Parameter	Result	Reference Range	Interpretation
WBC count	19.0 × 10⁹/L	4.0–11.0 × 10⁹/L	Leukocytosis
CRP	454 mg/L	<5 mg/L	Markedly elevated systemic inflammation
Procalcitonin	5 ng/mL	<0.5 ng/mL	Elevated suggestive of bacterial sepsis
Troponin-T	1,433 pg/mL	<14 pg/mL	Elevated myocardial injury
Serum Creatinine	2.08 mg/dL	0.6–1.2 mg/dL	Acute kidney injury
Blood urea	80.9 mg/dL	10–40 mg/dL	Elevated renal dysfunction
SGOT (AST)	636 U/L	5–40 U/L	Transaminitis
SGPT (ALT)	600 U/L	7–56 U/L	Transaminitis
Total bilirubin	Elevated	0.3–1.2 mg/dL	Hepatic dysfunction
Blood culture	MRSA isolated	No growth	Positive infective endocarditis

Diagnosis

Based on the clinical, laboratory, and imaging findings, the patient was diagnosed with septic shock, MI secondary to septic coronary embolism, MRSA‑positive IE of the aortic valve, and severe aortic regurgitation.

Management

The patient was stabilized with vasopressor support, intubated, and immediately taken for coronary angioplasty. Balloon dilatation and stenting of the posterior descending artery were performed with restoration of flow. Targeted antibiotic therapy was initiated with ceftazidime-avibactam, fluconazole, and teicoplanin. Given her worsening hemodynamics and echocardiographic evidence of vegetation with valve destruction, she was taken up for emergency aortic valve replacement.

**Figure 5:**
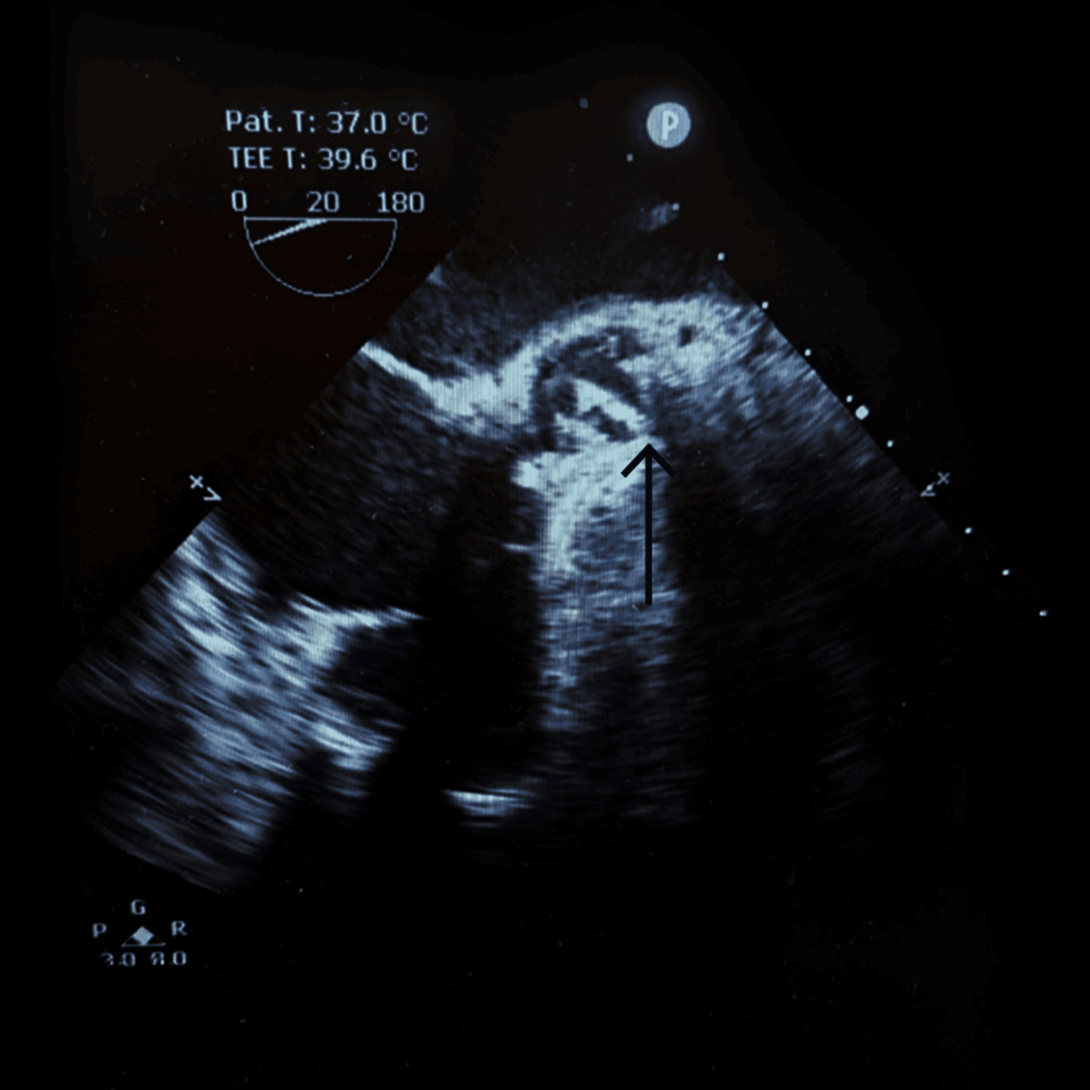
Intraoperative transesophageal image of the aortic valve in situ.

Intraoperatively, the pericardium was inflamed and densely adherent to the epicardium, with multiple pus-like collections. After initiating cardiopulmonary bypass, it was noted that the aortic valve had vegetations on the right coronary cusp and structural damage consistent with severe regurgitation. The aortic valve was excised and replaced with a St. Jude’s Regent mechanical prosthesis (size 21), which was confirmed by the intraoperative transesophageal echocardiography showing the aortic valve in situ (Figure 5).

Postoperative course

The patient was monitored in the intensive care unit postoperatively. She remained hemodynamically stable and was extubated on postoperative day two. Repeat echocardiography confirmed a well-seated, functioning prosthetic valve. Blood cultures turned sterile postoperatively. MRSA was found to be sensitive to vancomycin, which was continued. Renal and liver functions gradually normalized. Table 2 presents the timeline of the 11-day hospital course of the postpartum female with MRSA IE, septic coronary embolism, and severe aortic regurgitation.

**Table 2:** Timeline of the 11-day hospital course of a postpartum female with MRSA infective endocarditis and aortic valve replacement. BP = blood pressure; WBC = white blood cell count; CRP = C-reactive protein; MRSA = methicillin-resistant *Staphylococcus aureus*; ICU = intensive care unit; PDA = posterior descending artery

Day(s)	Clinical event	Key details/Interventions
1	Admission	Chest pain, dyspnea, intermittent fever; hypotension (BP 83/50 mmHg); labs: WBC 19 × 10⁹/L, CRP 454 mg/L, procalcitonin 5 ng/mL, Troponin-T 1,433 pg/mL; MRSA blood culture positive; echo: aortic valve vegetation (1.3 × 0.5 cm), severe aortic and tricuspid regurgitation; CT chest: pulmonary edema
1–2	ICU stabilization	Intubation, vasopressor support; initiation of antibiotics: ceftazidime-avibactam, fluconazole, teicoplanin
2	Coronary intervention	Coronary angiography: thrombotic occlusion of PDA and posterolateral branch; balloon angioplasty and stenting performed
3–4	ICU supportive care	Ongoing monitoring; renal and hepatic support; management of multi-organ dysfunction
4	Emergency aortic valve replacement	Intraoperative: inflamed pericardium with pus, vegetations on the right coronary cusp; valve replaced with St. Jude’s mechanical prosthesis (size 21)
5–7	Postoperative ICU care	Extubation on day 6; hemodynamic monitoring; repeat echo: well-functioning prosthetic valve
8–11	Step-down and recovery	Continuation of vancomycin, renal and hepatic functions normalizing; blood cultures sterile; gradual mobilization
11	Discharge	Stable condition; instructions for oral anticoagulation and antihypertensives; follow-up under cardiology

Outcome

The patient showed continuous improvement and was discharged on postoperative day 11. On the third-month follow-up, she was asymptomatic and continued on oral anticoagulation and antihypertensive therapy under regular cardiology supervision.

## Discussion

IE remains a critical cardiac condition with highly variable presentations and a complex clinical course. Its classic signs include fever, new or changing murmurs, and vascular or immunologic phenomena [1]. However, atypical presentations such as ACS or MI are rare, making early recognition particularly challenging [2]. In the present case, a postpartum woman with no prior cardiac history presented with features mimicking MI, later diagnosed as MRSA-associated IE of the aortic valve with septic embolism.

The association between IE and MI is uncommon and typically occurs through mechanisms such as coronary artery embolism from vegetations, compression from periannular abscesses, or septic arteritis. Coronary embolism, though rare, should be considered a possible cause of acute MI in patients with atrial fibrillation, prosthetic heart valves, or IE. While embolic events occur in nearly half of IE cases, coronary embolism accounts for only about 2-3% [7]. In the present case, coronary angiography revealed thrombus in the posterior descending and posterolateral arteries, which, in the absence of atherosclerotic disease, supports septic embolism as the cause of infarction. This is supported by previous case reports and reviews identifying coronary embolism as a rare but lethal complication of left-sided IE [8].

Postpartum IE is rare but well-documented. The annual incidence of IE has steadily risen from 5-7 cases per 100,000 person-years between 1970 and 2000 to approximately 15 cases per 100,000 in the U.S. by 2011 [4]. Pregnancy and the puerperium represent a state of immunomodulation that can predispose women to infections. Risk factors such as surgical delivery, prolonged catheterization, epidural anesthesia, and bacteremia increase susceptibility [3]. The current case aligns with prior studies where symptom onset was overlooked during pregnancy and exacerbated post-delivery, highlighting the challenges in timely diagnosis and management of postpartum IE [4].

MRSA is recognized as a particularly virulent pathogen in IE, frequently associated with rapid valve destruction, systemic embolization, and poor prognosis [1]. Early diagnosis of IE is crucial and begins with clinical suspicion in patients presenting with fever or sepsis, especially those with underlying risk factors such as prosthetic valves or intravenous drug use. Confirmation is supported by echocardiographic evidence of vegetations and positive blood cultures for typical organisms [5]. While ECG findings are often normal, they may help exclude other cardiac conditions or reveal complications such as MI [5]. In the present case, the aggressive nature of MRSA infection led to severe aortic regurgitation requiring urgent surgical intervention.

MRSA IE in postpartum women is exceedingly rare and carries significant maternal and fetal risk. Although the overall incidence of IE in pregnancy is estimated at 1 in 100,000 pregnancies, it is associated with high maternal (22.1%) and fetal (14.7%) mortality [4]. Pulmonic valve involvement accounts for ≤2% of all IE cases, and isolated native pulmonic valve MRSA IE is even more uncommon. In a 2020 case report, Navarrete et al., upon reviewing the PubMed literature, identified their case as the second documented instance of isolated native pulmonic valve MRSA IE, with the first also reported in a 19-year-old postpartum [9,10]. Risk factors for IE in the peripartum period include intravenous drug use, congenital heart disease, and immunological or mucosal changes associated with pregnancy and delivery. Due to its rarity and severity, early diagnosis and prompt treatment with appropriate antibiotics and surgical intervention when necessary are crucial in managing such cases [9].

Differentiating MI caused by IE from other cardiac conditions can be challenging due to overlapping symptoms. Patients with IE may present with chest pain, fever, elevated inflammatory markers, and high troponin levels, features that are also common in ACS and myocarditis. Although ECGs may show ST-segment elevations resembling a typical MI, the specific pattern of these changes, combined with the overall clinical context, can help point toward IE as the underlying cause [2]. Fever, signs of systemic infection, and embolic phenomena, particularly in patients without traditional cardiovascular risk factors, should prompt consideration of IE. Although MI resulting from septic emboli in IE is rare, it carries a high risk of morbidity and mortality. In such cases, early coronary angiography is essential for confirming the diagnosis and guiding appropriate treatment, especially in patients presenting with elevated troponin levels or cardiac dysfunction [11]. In our case, septic coronary embolism from IE led to MI, with overlapping symptoms and ECG findings that made diagnosis challenging.

ECG played a pivotal role in the diagnosis. Detection of mobile vegetation on the aortic valve with concurrent severe regurgitation warranted surgical management, consistent with current guidelines advocating early valve replacement in complicated IE [11]. Studies have emphasized that prompt surgical intervention improves survival in patients with left-sided IE complicated by embolism, heart failure, or persistent infection [5].

Aortic valve replacement in the setting of active endocarditis remains technically demanding due to inflamed and friable tissues. Intraoperative findings in this patient, including vegetations, torn leaflets, and pericardial adhesions, highlight the destructive nature of MRSA IE. The use of a mechanical prosthesis was appropriate given the patient’s age and absence of contraindications. Studies comparing outcomes between mechanical and bioprosthetic valves in IE suggest similar infection recurrence rates, with mechanical valves favored in younger patients [12,13].

The patient’s favorable postoperative course reinforces the importance of a multidisciplinary approach. Timely resuscitation, accurate diagnosis, early revascularization, appropriate antibiotics, and definitive surgical intervention collectively contributed to recovery. This case underscores the need for clinicians to maintain high suspicion for IE in patients with systemic symptoms and cardiac findings, even in the context of atypical presentations such as MI. However, being a single case, generalizability is limited. Long-term outcomes and pathogen-specific resistance data were not explored, which could have added depth to the clinical insights.

## Conclusions

This case highlights the rare but clinically significant presentation of IE as an acute MI, particularly in a postpartum woman without a known cardiac history. MRSA can lead to rapid valvular destruction, systemic embolism, and multiorgan dysfunction. Early suspicion, timely diagnostic imaging, including echocardiography, and blood cultures are critical for accurate diagnosis. A multidisciplinary approach comprising hemodynamic stabilization, percutaneous coronary intervention, targeted antimicrobial therapy, and urgent surgical valve replacement can significantly improve outcomes. Clinicians should maintain a high index of suspicion for IE in any febrile patient presenting with cardiac symptoms, especially those in immunomodulatory states such as the postpartum period. Prompt recognition and intervention can be lifesaving in such complex and rapidly deteriorating clinical scenarios.
